# Rapid Assessment of Bio-distribution and Antitumor Activity of the Photosensitizer Bremachlorin in a Murine PDAC Model: Detection of PDT-induced Tumor Necrosis by IRDye® 800CW Carboxylate, Using Whole-Body Fluorescent Imaging

**DOI:** 10.1007/s11307-024-01921-1

**Published:** 2024-06-18

**Authors:** Roisin McMorrow, Henriette S. de Bruijn, Ivo Que, Debra C. Stuurman, Corrina M.A. de Ridder, Michail Doukas, Dominic J. Robinson, Laura Mezzanotte, Clemens W.G.M. Lowik

**Affiliations:** 1 Department of Radiology and Nuclear Medicine, Erasmus Medical Centre, Rotterdam, The Netherlands; 2 Department of Molecular Genetics, Erasmus Medical Centre, Rotterdam, The Netherlands; 3 Department of Otorhinolaryngology and Head and Neck Surgery, Erasmus MC Cancer Institute, University Medical Center Rotterdam, Rotterdam, The Netherlands; 4 Department of Urology, Erasmus Medical Centre, Rotterdam, The Netherlands; 5 Department of Pathology, Erasmus Medical Centre, Rotterdam, The Netherlands

**Keywords:** Photodynamic therapy, PDT, PDAC, Necrosis, Photosensitizer, PS, Bremachlorin, Fluorescence, Whole-body imaging, Optical imaging

## Abstract

Photodynamic therapy (PDT) is a light-based anticancer therapy that can induce tumor necrosis and/or apoptosis. Two important factors contributing to the efficacy of PDT are the concentration of the photosensitizer in the tumor tissue and its preferential accumulation in the tumor tissue compared to that in normal tissues. In this study, we investigated the use of optical imaging for monitoring whole-body bio-distribution of the fluorescent (660 nm) photosensitizer Bremachlorin *in vivo*, in a murine pancreatic ductal adenocarcinoma (PDAC) model. Moreover, we non-invasively, examined the induction of tumor necrosis after PDT treatment using near-infrared fluorescent imaging of the necrosis avid cyanine dye IRDye®-800CW Carboxylate. Using whole-body fluorescence imaging, we observed that Bremachlorin preferentially accumulated in pancreatic tumors. Furthermore, in a longitudinal study we showed that 3 hours after Bremachlorin administration, the fluorescent tumor signal reached its maximum. In addition, the tumor-to-background ratio at all-time points was approximately 1.4. *Ex vivo*, at 6 hours after Bremachlorin administration, the tumor-to-muscle or -normal pancreas ratio exhibited a greater difference than it did at 24 hours, suggesting that, in terms of efficacy, 6 hours after Bremachlorin administration was an effective time point for PDT treatment of PDAC. *In vivo* administration of the near infrared fluorescence agent IRDye®-800CW Carboxylate showed that PDT, 6 hours after administration of Bremachlorin, selectively induced necrosis in the tumor tissues, which was subsequently confirmed histologically. In conclusion, by using *in vivo* fluorescence imaging, we could non-invasively and longitudinally monitor, the whole-body distribution of Bremachlorin. Furthermore, we successfully used IRDye®-800CW Carboxylate, a near-infrared fluorescent necrosis avid agent, to image PDT-induced necrotic cell death as a measure of therapeutic efficacy. This study showed how fluorescence can be applied for optimizing, and assessing the efficacy of, PDT.

**Supplementary Information:**

The online version contains supplementary material available at 10.1007/s11307-024-01921-1.

## Introduction

Photodynamic therapy (PDT) is a clinically approved and minimally invasive cancer therapy that involves the activation of a photosensitizer (PS) using light at a specific wavelength. In addition to their therapeutic abilities, PSs can act as fluorophores. These fluorescent properties of PSs can be utilized in dosimetry and bio-distribution studies and can also be used for diagnosis [1]. Knowing the amount of PS in the tumor before PDT illumination can help in defining an efficient drug/light interval and adjusting the light delivery conditions since it has been shown that this can help to improve the response rate [2]. PSs can be used for many imaging applications, but they do not necessarily reflect the tumor’s response to PDT [2].

Bremachlorin is a chlorin-based, second-generation, PS also known as Radachlorin®. It specifically accumulates in tumors, is rapidly excreted from normal tissue and alone shows low toxicity [3]. The fluorescent emission spectrum of Bremachlorin shows a peak at approximately 666 nm in the red light spectrum [3]. Bremachlorin mediated PDT has been investigated in different cancer types such as colon, colorectal, lymphoma, lung and oral squamous cell carcinomas [3–8]. In addition, Bremachlorin PDT has been tested in a phase II clinical trial for treating basal cell carcinomas [9]. PDT induced cell death can lead to an immune response through primary or secondary necrotic cells that release intracellular molecules. These molecules are known as danger associated molecular patterns (DAMPs) and are typically recognized by the innate immune cells that can lead to the activation of the immune system [10]. Previously, we have shown that combining Bremachlorin mediated PDT with immunotherapy enhances treatment response [5, 8]. In these previous studies, Bremachlorin PDT was typically performed 6 hours after the injection of the PS [5, 8].

PDT induces direct tumor cell death through the production of reactive oxygen species (ROS), which can induce necrosis and / or apoptosis [11]. In addition to these main forms of cell death, PDT has been reported to induce autophagy and other forms of programmed necrotic death, including paraptosis, necroptosis, ferroptosis, pyroptosis, parthanatos and mitotic catastrophe [12]. In addition, if apoptotic cells are not removed in a timely manner by macrophages, the cells become necrotic, which is known as secondary necrosis and is the natural outcome of the complete apoptotic program [13]. Therefore, tissue necrosis can occur as the end result of many types of cell death, rendering necrosis as an interesting and relevant imaging target for assessing therapeutic efficacy [14]. Assessing cell death through imaging can contribute to determining the effectiveness of PDT [2].

Previously, we demonstrated that the near-infrared cyanine dye IRDye®-800CW Carboxylate (referred to hereafter as 800CW Carboxylate) could be used to indicate an increase in necrotic cell death after chemo- and radiotherapy in mice bearing murine breast and T-cell lymphoma tumors, indicating that this cyanine can be used as a non-invasive necrosis targeting imaging agent [15, 16]. *In vitro* studies have shown that 800CW Carboxylate specifically binds to denatured intracellular cytoplasmic proteins in necrotic cells, after cell membrane integrity is disrupted [16]. Unconjugated 800CW *in vivo* shows the majority of the dye clears in under 24 hours after i.v injection, has shown no evident toxicity in rats and has been considered safe to use in humans [17–19]. Moreover, 800CW Carboxylate emits light in the near-infrared region at 800 nm allowing for deep tissue penetration, rendering it beneficial as an optical imaging agent for *in vivo* application in small animals.

Different PSs have been examined for PDT treatment of pancreatic cancer *in vivo,* but Bremachlorin has not yet been investigated. The most common form of pancreatic cancer is pancreatic ductal adenocarcinoma (PDAC) [20]. In this study, we used mice transplanted with murine PDAC cells derived from a spontaneous, genetically engineered, mouse model termed KPCY [21]. In the present study, we investigated the whole-body distribution, *in vivo* and *ex vivo*, of the PS Bremachlorin in PDAC tumors by utilizing its fluorescent properties, as a tool to determine an effective time point for PDT treatment. In addition, we investigated 800CW Carboxylate, a necrosis avid fluorescent agent, as a tool to evaluate PDT-induced cell death in PDAC tumors.

## Methods

### Cell Culture

The murine pancreatic ductal adenocarcinoma (PDAC) tumor (KPC) cell clone type 2838c3 was derived from KPCY mice [21]. We kindly received these cells from Dr. Stanger at the University of Pennsylvania. PDAC (2838c3) cells were cultured in Dulbecco’s modified Eagle’s medium (DMEM) (Gibco) supplemented with 10% FBS and 1% penicillin-streptomycin (PenStrep) at 37°C with 5% CO_2_. The cells were regularly tested for mycoplasma contamination.

### Mouse Model

Six- to eight-week-old female BALB/c nude mice (BALB/cOlaHsd-Foxn1nu, Envigo, The Netherlands) were subcutaneously (s.c.) bilaterally injected, with 30μl of 3x10^5^ tumor cells in PBS (PDAC clone type 2838c3) under general anesthesia (2.5% isoflurane/O_2_). Tumor growth was measured with calipers, and the three orthogonal diameters of the tumors were measured. The tumor volume was estimated using the formula Vt=D1*D2*D3*PI/6.

### Reagents Used

Bremachlorin (Radapharma International B.V., The Netherlands) was protected from light when handled, and the reagent was divided into aliquots, which were stored at 4°C. IRDye®-800CW Carboxylate (LI-COR Biosciences, Lincoln, NE) is a commercially available fluorescent near-infrared dye. Briefly, 2 nmol IRDye®-800CW Carboxylate aliquots were prepared in PBS.

### *In Vivo* Bremachlorin Uptake

Mice were randomly assigned to one of the three treatment groups: 6 hours Bremachlorin; repeated-imaging 24 hours Bremachlorin; and control when tumors reached a volume of 60-80 mm^3^. This volume was reached 13 days after inoculation of the tumor cells (Supplementary Fig. 3a). The mean tumor treatment volumes were not significantly different between the groups (Supplementary Fig. 3c). Bremachlorin was administered at 20 mg/kg, 3.5 mg/ml, via the i.v. route via the tail vein to the Bremachlorin receiving groups (*n*=8) [3, 5–8]. 6 hours Bremachlorin group (*n*=4) were housed in subdued light conditions for 6 hours. Repeated-imaging 24 hours Bremachlorin group were kept in subdued lighting for 24 hours but were imaged at four different time points: 3, 4.5, 6 and 24 hours. Control mice (*n*=4) received nothing and were housed in subdued lighting conditions for 6 hours before imaging. The animals were imaged on an IVIS Spectrum imager (Revvity, Waltham, MA) under general anesthesia (2.5% isoflurane/O_2_). The animals were imaged dorsally and on both the right and left sides, using an FOV C, medium binning, excitation at 605 nm and emission 660 nm with 5 seconds of exposure. Signal quantification was performed by drawing regions of interest (ROIs) around the tumor. The general background signal was corrected by subtracting the signal from the same sized ROI placed on the animal’s back and used for each repeated time point using Living Image® 4.7.3 software (Revvity). For the tumor-to-background ratio (TBR), the background skin signal was measured using the same sized ROI (of the tumor) placed next to the animal’s tumor and used for each repeated time point. The values represent the average radiant efficiency [p/s/cm^2^/sr]/[μW/cm^2^].

After imaging, the animals were sacrificed by cervical dislocation. The tumors and specific organs, liver, spleen, stomach, small intestine, kidney, muscle, skin of the tumor and pancreas, were removed, weighed and imaged on an IVIS Spectrum imager (Revvity) with the same settings as explained above. The tumors were cut in half, one part for histology analysis and the other part for fluorescence bio-distribution measurements. The samples were then flash frozen with liquid nitrogen and stored at -80°C overnight before homogenization.

### *Ex Vivo* Bremachlorin Bio-distribution

For fluorescence quantification, the specific organs and tumors were disrupted using a TissueLyser II system (Qiagen, Venlo, The Netherlands), using precooled Eppendorf holders, 5-mm stainless steel beads, and 1x RIPA buffer (50 mM Tris-HCl pH 7.5, 150 mM NaCl, 1 mM EDTA, 1% Triton X-100, 0.1% SDS). A dilution series of Bremachlorin in RIPA buffer was performed to determine the linear range of the photosensitizer. 150 μl of homogenate solution was loaded into a microplate, 96 well, μclear, Schwarz high binding (cat. 665097 Greiner plates) and measured on a plate reader (Spectramax, Molecular Devices, USA) with excitation 605 nm and emission 660 nm settings.

### *Ex Vivo* Bremachlorin Histology Analysis

Tumors from the 6 hour Bremachlorin group (*n*=4) and the control group (*n*=4) were subjected to fluorescence microscopy and histological analysis. The 6 hour time point was chosen because previous studies used this time point for PDT [5, 8]. Freshly frozen tumor tissue was cut into sections of 20 μm in width on starfrost glass slides, using a cryostat and stored at -80°C. The samples were shielded from light as much as possible. For Bremachlorin fluorescence confocal imaging, the sections were thawed and imaged using a Leica SP5 confocal microscope with 405 nm excitation, a band pass filter of 650-700 nm detection and 10x and 20x objectives. Immediately after, the sections were subjected to immunofluorescence analysis. The sections were stained with an anti-CD31 antibody and Alexa-Fluor 488 (BioLegend cat# 102514, 1:200) overnight at 4°C in a humidified chamber. The next day, the slides were carefully rinsed in PBS, incubated for 10 minutes with Hoechst 3342 (1:1000 PBS) and covered with glycerol (diluted in PBS, 1:3) and a glass cover slip and sealed with transparent nail polish. The slides were then stored at 4°C and imaged within 4 days of mounting. The slides were imaged with the same confocal microscope (Leica SP5) with a bright-field and 405 nm excitation and with a band pass of 430-470 nm detection for Hoechst and 488 nm excitation and with a band pass of 520-550 nm for CD31 detection. Images were analyzed for CD31 and Bremachlorin co-localization using the Coloc 2 plug-in with ImageJ-Fiji. This generated a Spearman rank correlation coefficient from the images derived from two tissue samples from each group: the control and 6 hour Bremachlorin groups. Two or three images were taken per sample. The coverslips from the same slides were carefully removed, and the sections were stained with hematoxylin and eosin (H&E) using a standard protocol and imaged on a NanoZoomer (Hamamatsu, Japan).

### *In Vivo* Photodynamic Therapy (PDT)

Mice were randomly assigned to one of two treatment groups: the Bremachlorin-PDT group or the control group, when the tumor volume reached 60-80mm^3^. This volume was reached on average between days 14-16 after inoculation of the tumor cells (Supplementary Fig. 3b). The mean tumor treatment volumes were not significantly different between the groups (Supplementary Fig. 3d). Bremachlorin was administered at 20 mg/kg, 3.5 mg/ml, via the i.v. route via the tail vein to the PDT receiving group (*n*=5), and the mice were housed in subdued light conditions for 6 hours. Preventative pain relief agent (buprenorphine 0.1 mg/kg, 6 μl/gram) was administered s.c. both pre- and post-PDT. The illumination was performed under general anesthesia (2.5% isoflurane/O_2_). Mice were placed on a temperature-controlled stage, and the tumor was positioned loosely to the side of the mouse to ensure that the illumination beam contained only the tumor and the skin, the rest of the mouse was shielded with black paper. The tumors were then trans-dermally illuminated with a 662 nm laser (Millennia Pro solid-state CW 532 pump laser, Spectra Physics, Utrecht, The Netherlands) combined with a Matisse dye laser with DCM Dye (Syrah, Grevenbroich, Germany) and a frontal light diffuser (Medlight SA, Ecublens, Switzerland) at 116 mW/cm^2^ to a fluence of 116 J/cm^2^ (described previously [5]). Mice in the control group (*n*=5) received no drug or pain relief and were housed in subdued light conditions for 6 hours.

### *In Vivo* 800CW Carboxylate Uptake

IRDye®-800CW Carboxylate (100ul of 2 nmol in PBS, LI-COR Biosciences) was i.v. administered via the tail vein 24 hours after PDT or control treatment. Imaging was performed under general anesthesia (2.5% isoflurane/O_2_) 24 hours after receiving 800CW Carboxylate administration using an IVIS Spectrum imager (Revvity). The mice were imaged dorsally and on both the right and left sides, using an FOV C, medium binning, excitation at 710 nm and emission 820 nm with 10 seconds of exposure. Signal quantification was performed by drawing regions of interest (ROIs) around the tumor. The background signal was corrected by subtracting the signal from the same-sized ROI placed next to the tumor on the mouse using Living Image® 4.7.3 software (Revvity). The values represent the average radiant efficiency [p/s/cm^2^/sr]/[μW/cm^2^].

### Schematic of PDT Treatment and 800CW Carboxylate Administration and Imaging

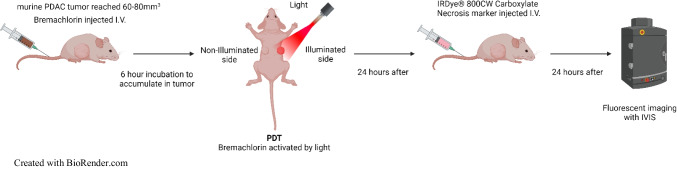



### *Ex Vivo* Histology Analysis of 800CW Carboxylate

After 800CW Carboxylate imaging, the animals were sacrificed by cervical dislocation. The tumors were removed, flash frozen with liquid nitrogen and stored at -80°C. The frozen tumor tissues were cut at the cryostat into 10 μm thick sections, mounted on glass slides and stored at -80°C until use. For 800CW Carboxylate fluorescent imaging, the slides were imaged on an Odyssey® M Imaging System at 5 μm resolution with the 800 nm fluorescent channel (ex 785 nm/em 820 nm) (LI-COR Biosciences). Consequent sections were stained with hematoxylin and eosin (H&E) using a standard protocol and imaged on an NanoZoomer (Hamamatsu). The H&E stained sections were assessed blindly by a pathologist to annotate the tumor tissue and necrotic tissue. The percentage of the necrotic area was calculated by measuring the ratio of the necrotic area or fluorescent area to the tumor area and multiplying by 100. The H&E images were analyzed using NDP view 2 software (Hamamatsu), and the fluorescence images were analyzed using Empiria Studio software (LI-COR Biosciences).

### Animal Protocol

The animal protocol was approved by the Institutional Animal Care and Bioethics Committee of the Erasmus MC, Rotterdam, The Netherlands (approval number AVD1010020209868 and work package (WP) number SP2200243). The experiments were conducted in accordance with the national guidelines (National CCD license 209868) and regulations established by the Dutch Experiments on Animals Act (WoD) and by the European Directive (2010/63/EU) on the Protection of Animals used for scientific purposes.

### Data Analysis

All the statistical analyses were performed using GraphPad Prism 9.0 software (GraphPad Software, San Diego, CA, USA). For both the Bremachlorin and 800CW Carboxylate uptake *in vivo* fluorescence and for the tumor-to-background ratio, one-way ANOVA with Tukey’s multiple comparisons test was used. For the *ex vivo* bio-distribution two-way ANOVA with Bonferroni’s multiple comparison test was used. Differences were considered significant when * *p*<0.05, ** *p*<0.01, *** *p*<0.005, and **** *p*<0.0001.

## Results

### *In Vivo* Optical Imaging of the Photosensitizer Bremachlorin

**Fig. 1:**
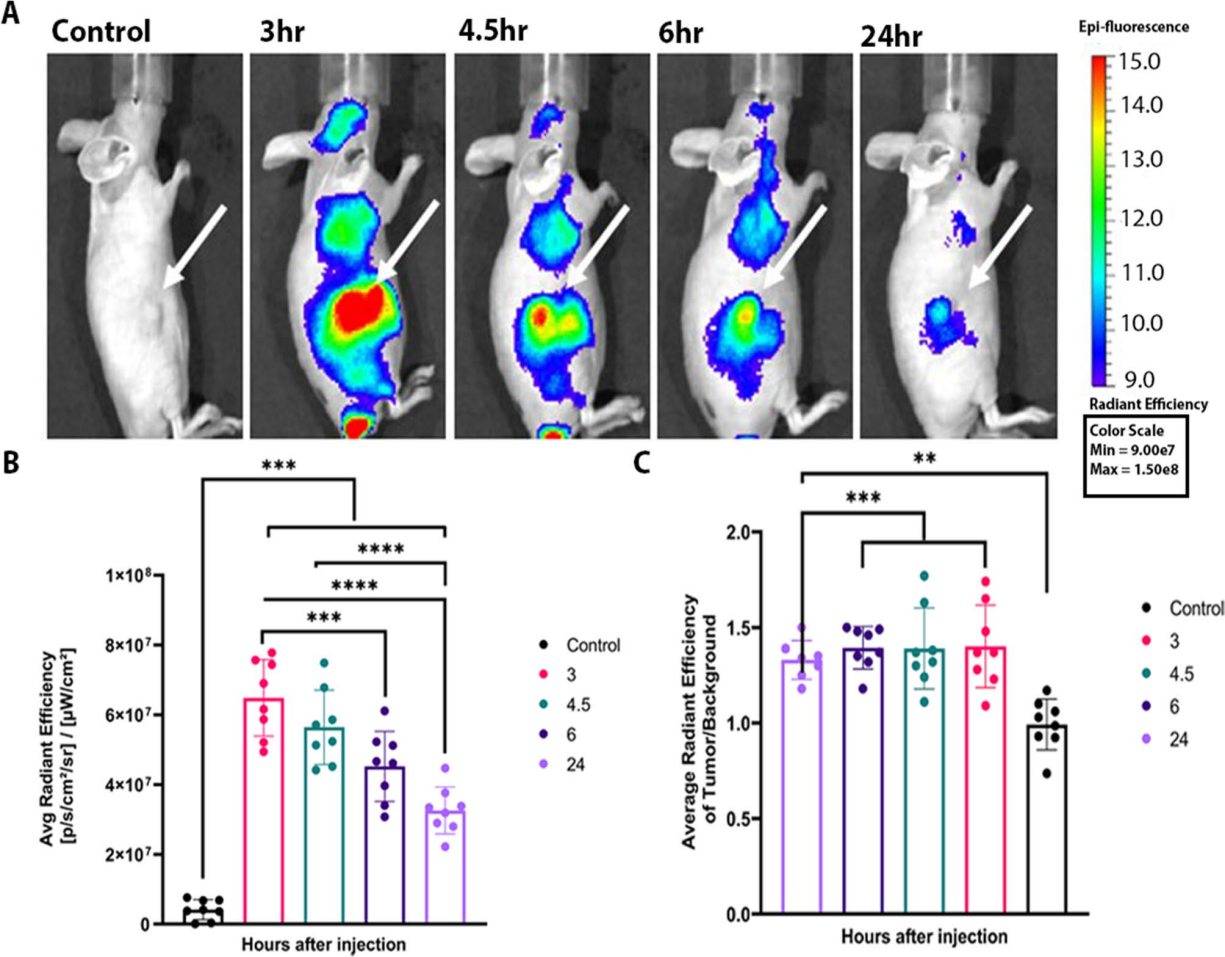
*In Vivo* Optical Imaging of the Photosensitizer Bremachlorin. **A** Representative fluorescence whole-body images of mice bearing PDAC lesions, right dorsal side, tumors taken longitudinally. **B** Bar graph representing the corrected fluorescent signal (average radiant efficiency) of Bremachlorin in the tumor over time, the control group did not receive Bremachlorin (*n*=8 for the control group and 3-,4.5-,6-, and 24-hour groups). **C** Bar graph representing the tumor-to-background ratio (TBR) of the fluorescent signal (average radiant efficiency) of the tumor divided by the fluorescent signal (average radiant efficiency) of the representative surrounding tissue (*n*=8 for the control group and 3-,4.5-, and 6-hour groups, except for the 24-hour group *n*=7 due to missing data). All p- values shown were determined by one-way ANOVA using Tukey’s multiple comparisons test, ** *p*<0.01, *** *p*<0.005, **** *p*<0.0001. The data are presented as the means ± SDs

After administration, the fluorescence of Bremachlorin in PDAC tumor-bearing mice was imaged over time (Fig. 1a). At all-time points, the fluorescent signal of the tumor was significantly different from that of the controls, which did not receive the PS (*p*<0.0001) (Fig. 1b). At the 3-hour time point, the fluorescent signal was greater than the other time points, with a significant difference relative to 6- and 24-hour (*p*=0.0007 compared to 6-hour and *p*<0.0001 for 24-hour). No difference was found between the 3- and 4.5-hour groups (*p*=0.3325); or the 4.5- and 6-hour groups (*p*=0.1035). The 4.5-hour group showed a significant difference from the 24-hour group (*p*<0.0001). There was no significant difference between the 6- and 24- hour groups (*p*=0.0503). The tumor-to-background ratio (TBR), did not significantly differ among the four time points but did significantly differ from that of the controls (*p* value =0.0002 for 3-,4.5- and 6-hour and *p*=0.0029 for 24-hour) (Fig. 1c).

### Assessing the Photosensitizer Bremachlorin *Ex Vivo*

**Fig. 2:**
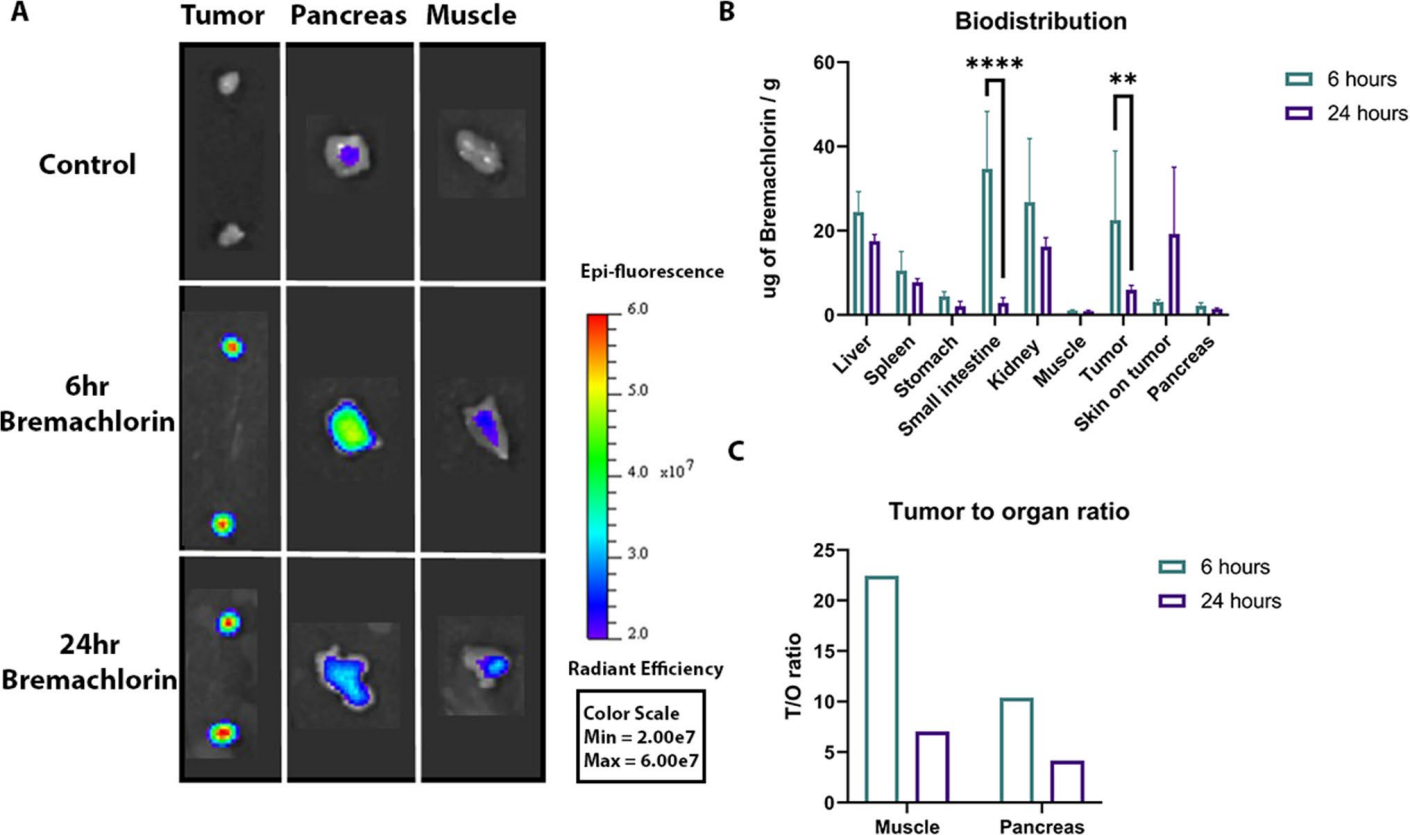
Assessment of the Photosensitizer Bremachlorin *Ex Vivo*. **A** Representative fluorescence images of tumors, pancreases and muscles *ex vivo* at 6 and 24 hours after Bremachlorin injection and the control group (that did not receive Bremachlorin). **B** Bar graph representing the amount of Bremachlorin (μg) per gram (g) of tissue calculated from the fluorescent signal of the homogenized tissue (at each time point, *n*=4 for organs and *n*=8 for tumors). **C** Bar graph representing the tumor-to-organ (T/O) ratio of either the muscle or pancreas from the values plotted in (B). All p- values were determined by two-way ANOVA using Bonferroni multiple comparisons test; ** *p*<0.01, **** *p*<0.0001. The data are presented as the means ± SDs

The distribution of Bremachlorin in tumors and organs was also imaged *ex vivo* on a 2D planar view (Fig. 2a and Supplementary Fig. 1). After homogenizing the organs to measure the amount of Bremachlorin, we observed more fluorescence at 6-hour than at 24-hour (*p*=0.0019 Fig. 2b). We observed no difference in fluorescence between the organs at 6- and 24-hour except for the small intestine, which showed a greater fluorescence at 6-hour than at 24-hour (*p*<0.0001). The tumor-to-organ (T/O) ratios for tumor-to-muscle and tumor-to-pancreatic tissue were greater at 6-hour than at 24 hour (Fig. 2c).

### Investigating the Microscopic Distribution of Bremachlorin in PDAC Tumors

**Fig. 3:**
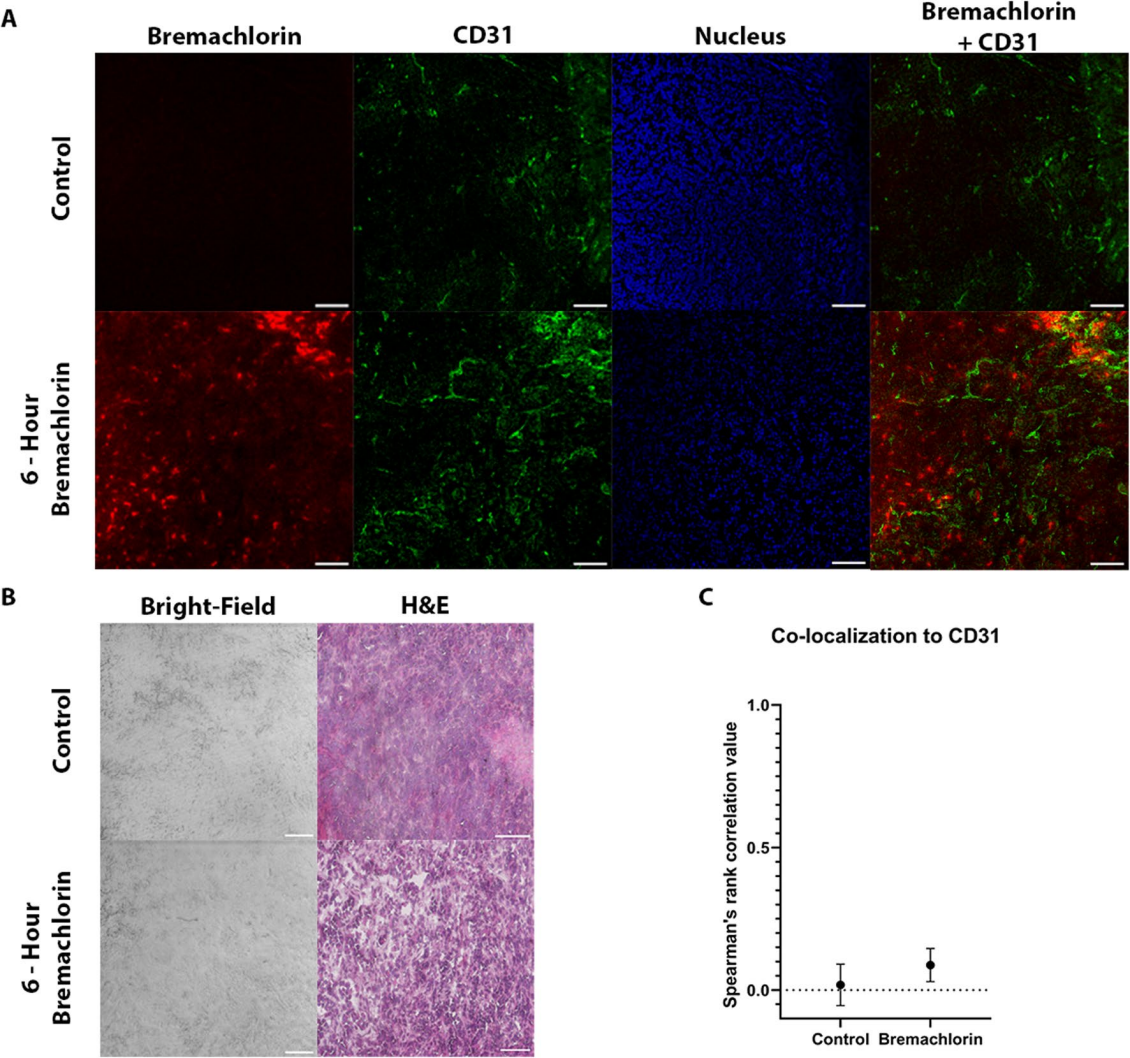
Investigating the Microscopic Distribution of Bremachlorin in PDAC Tumors. **A** Representative fluorescence confocal images of 6-hour Bremachlorin (red) and control (that did not receive Bremachlorin) tumor tissues (*n*=1 for each condition). Tumor tissue was stained with the endothelial marker CD31 (green) and the nuclear marker Hoechst (blue). **B** Representative bright-field confocal image of the fluorescence images shown in (A) and scanned H&E-stained tumor tissue. **C** The graph represents the co-localization of Bremachlorin with CD31-stained vascular endothelial cells calculated by Spearman’s rank correlation value. The values are presented as the average of 2 tumor samples per group, with 2 or 3 images taken per sample. All images in this figure are at 20x magnification, and scale bars indicate 100 μm. The data are presented as the means ± SDs

Fluorescence microscopy of the tumor tissue samples showed that Bremachlorin was taken up by the tumor tissue in a non-homogenous manner (Fig. 3a). A greater signal of Bremachlorin fluorescence was observed in some parts of the tissue than in others. We stained for H&E to look at histological features (Fig. 3b). Co-localization analysis between Bremachlorin and CD31 revealed no correlation (Spearman’s rank correlation value 0.09) (Fig. 3c).

### *In Vivo* Optical Imaging of PDT-induced Cell Death with 800CW Carboxylate

**Fig. 4:**
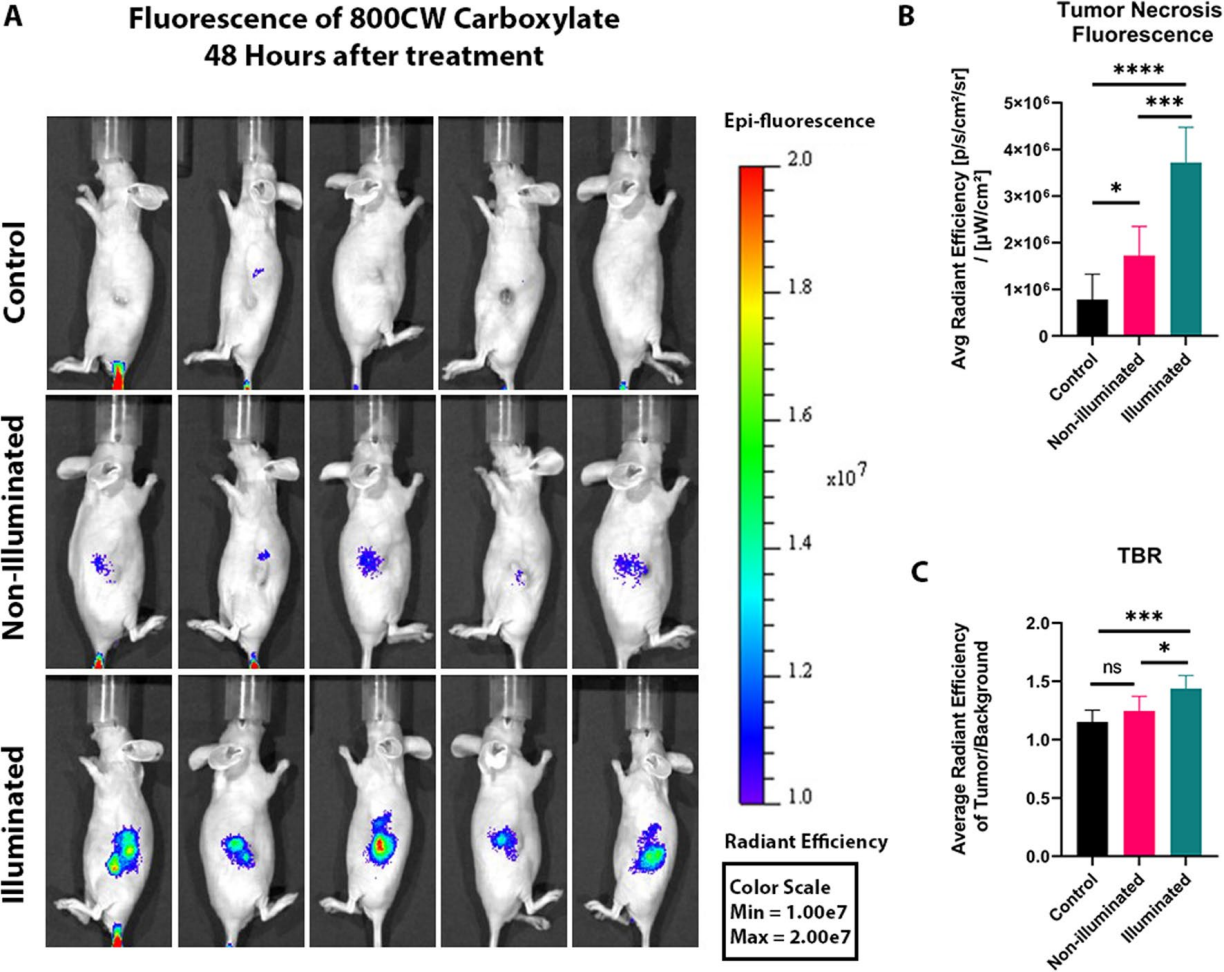
*In Vivo* Optical Imaging of PDT-induced Cell Death with 800CW Carboxylate. **A** Fluorescent whole-body imaging of PDAC tumor-bearing mice. All the mice shown were administered 800CW Carboxylate 24 hours prior to imaging. **B** Bar graph representing the corrected fluorescent signal (average radiant efficiency) of 800CW Carboxylate in tumors from the PDT-illuminated (*n*=5), PDT-non-illuminated (*n*=5) and control (*n*=10) groups. All the groups received 800CW Carboxylate, but the controls did not receive Bremachlorin. **C** Bar graph representing the tumor-to-background ratio of the fluorescent signal (average radiant efficiency) of the tumor divided by the fluorescent signal (average radiant efficiency) of the representative surrounding tissue. All p- values were determined by one-way ANOVA with Tukey’s multiple comparisons test; ns represents non-significant differences; * *p*<0.05, *** *p*<0.005, **** *p*<0.0001. The data are presented as the means ± SDs

All five PDT illuminated tumors showed a greater 800CW Carboxylate fluorescent signal compared to their non-illuminated tumors (*p* value = 0.0002) and control tumors (*p*<0.0001) (Fig. 4a-b). When comparing the TBR, the PDT-illuminated tumors were significantly different from the non-illuminated tumors (*p* value = 0.03) and the control tumors (*p*=0.0005) (Fig. 4c). The non-illuminated tumors presented a greater 800CW Carboxylate fluorescence signal than the controls did (*p* value = 0.03) (Fig. 4b). However, when comparing the TBR, no difference was detected (Fig. 4c).

### Assessment of *Ex Vivo* Cell Death with 800CW Carboxylate

**Fig. 5:**
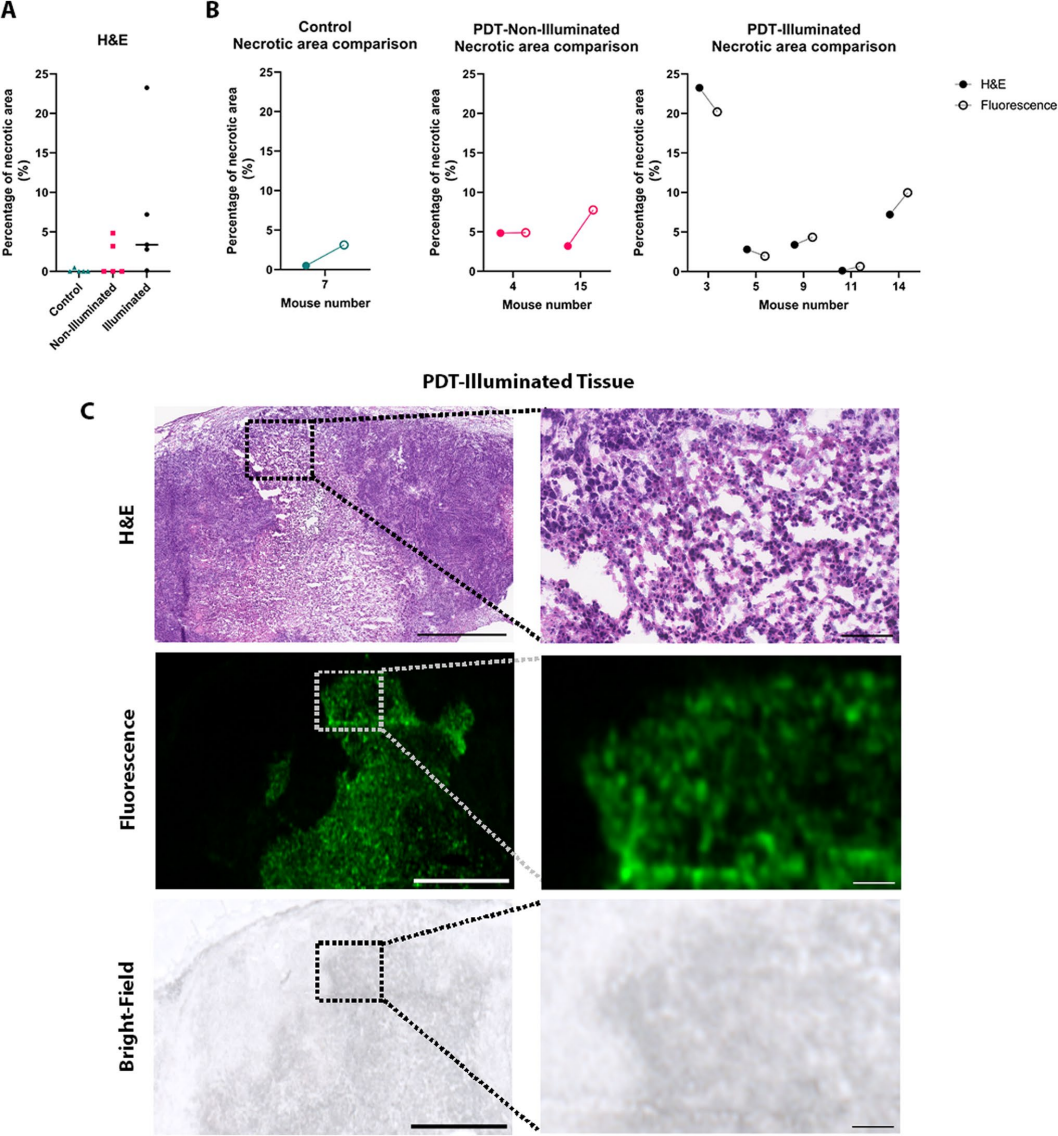
Assessment of *Ex Vivo* Cell Death with 800CW Carboxylate. **A** The graph represents the percentage of necrotic tissue (%) in H&E-annotated tumor slices determined by pathologist from the PDT-illuminated, PDT-non-illuminated and control groups (*n*=5 per group). **B** Graph representing the percentage of necrotic tissue (%) in H&E-annotated tumor slices (same as in figure A) compared to the percentage of necrotic tissue (%) in fluorescence images of the PDT-illuminated (*n*=5), PDT-non-illuminated (*n*=2) and control (*n*=1) groups. **C** Representative images of PDT-illuminated tumor tissue parallel slices of H&E-stained (top images), 800CW Carboxylate fluorescence (middle images), and bright-field of fluorescence images (bottom images). All images in this figure are shown with scale bars of 1mm (left) or 100 μm (right)

H&E staining of the tumors collected 48 hours after treatment revealed a greater percentage of necrotic tissue in the PDT-illuminated group than in the non-illuminated and control groups (Fig. 5a). All PDT-illuminated sections displayed necrotic tissue within the tumor however, one showed minimal damage. Quantification of the percentage of necrosis relative to the total tumor area revealed comparable levels of necrosis in pathologically assessed H&E sections and fluorescence image sections (Fig. 5b). When comparing the H&E stained tumor tissue to the 800CW Carboxylate fluorescence signal the annotated necrosis areas overlapped (Fig. 5c and Supplementary Fig. 2). In addition to the PDT-illuminated necrotic sections, two non-illuminated tumors and one control tumor also exhibited a small amount of necrosis which was also apparent in the fluorescence images (Supplementary Fig. 2).

## Discussion

In this study, we utilized, *in vivo*, whole-body, fluorescent imaging as a rapid assessment method for evaluating the efficacy of Bremachlorin PDT in pancreatic cancer for the first time. By utilizing the fluorescent properties of the PS Bremachlorin, we were able to determine an effective time point for PDT illumination in a PDAC tumor model *in vivo*. This was then verified *ex vivo* to determine the tumor distribution. We confirmed that 800CW Carboxylate can be used as a fluorescent necrosis imaging agent for assessing PDT-induced cell death.

Previously, our group used optical spectroscopy to quantitatively measure, *in vivo*, the intrinsic fluorescence spectrum of Bremachlorin in oral squamous cell carcinoma tissue [6]. This method can provide useful information on the optical properties of the PS but has limited spatial resolution. In our current study, we investigated the potential of using fluorescent imaging to non-invasively monitor the whole-body distribution of the fluorescent PS Bremachlorin in real time in a rapid and cost-effective way. We examined the distribution and accumulation of Bremachlorin in the tumor and surrounding area *in vivo.* To further examine this distribution, we performed *ex vivo* microscopic analysis of the tissue.

When comparing our findings with our previous quantification of Bremachlorin in oral squamous cell carcinoma, we observed a gradual decrease in signal from the tumor tissue starting from the 3-hour time point after administration [6]. The concentration of the PS helps determine an efficient drug/light interval to optimize the PDT response. The 3-hour time point showed the greatest fluorescent signal in the tumor but the TBR showed no difference at the four different time points (Fig. 1b and c). From this we predicted a higher potential of damage to normal tissue at the earlier time point of 3-hours, induced by Bremachlorin mediated PDT, due to the higher presence of Bremachlorin in the surrounding tissue or vasculature, whereas at the later time points, less but still enough PS was retained in the tumor tissue for the PDT effect. To investigate whether the concentration differed between the 6- and 24-hour groups, and to determine whether there was a better TBR, we measured the average fluorescence of the tumor *ex vivo*. Previously, our group reported a small increase in the T/O ratio in normal tissue at 24 hours after administration compared to that at earlier time points, which was not observed in our present analysis [7]. We found that there was a significantly greater amount of Bremachlorin at 6 hours, as well as a greater difference in the T/O ratio in the muscle- or in the normal pancreas-to-tumor ratio at 6 hours than at 24 hours. We concluded from this analysis that it was effective to continue using the 6-hour time point instead of the 24-hour time point, as the difference in the muscle- or pancreas-to-tumor ratio is important for minimizing off-target effects, and the overall concentration of Bremachlorin was greater.

The location of the PS can indicate what will be damaged after PDT [22]. Our fluorescence microscopy analysis revealed a heterogeneous distribution of the PS, which could be one indication of the variation in the site at which tissue necrosis was induced after PDT (Fig. 3a, Fig. 5c and Supplementary Fig. 2). The tumors were vascularized but disorganized with capillary-like vessels, and both of these features are typically found in tumors (Fig. 3a) [23]. From our analysis, we found no vascular co-localization with Bremachlorin 6 hours after administration (Fig. 3c).

800CW Carboxylate has been shown to be an effective necrosis marker for many different tumor models and for different treatment interventions, such as chemo- and radiotherapy, but to the best of our knowledge, this is the first study in which it has been used to assess PDT-induced cell death [15, 16]. We were provided crucial information on the efficacy of PDT using a necrosis imaging agent to assess post treatment effects in pancreatic cancer. Measuring the induction of cell death after cancer treatment *in vivo* can be an early indicator of its efficacy. The understanding of the complexities of cell death, and all of its different forms, has grown extensively over the last several decades [24]. Identifying a reliable and general cell death marker would be beneficial for assessing treatment efficacy. Using a tracer for tissue necrosis can lead to an interesting imaging target for such purposes, as necrosis is the result of many types of cell death [6], including apoptosis. If the formed apoptotic bodies fail to be cleared rapidly enough by tissue macrophages, secondary necrosis occurs [13], also known as late apoptotic/necrotic cell clearance, which means that a necrosis marker can also indicate this form of cell death [25]. Currently, in the clinic, there are no quantitative necrosis probes available.

Of course, necrosis in solid tumors is often linked to the aggressiveness of the tumor type due to poor vasculature quality, which reduces the availability of oxygen and nutrients in the center of the tumor [26, 27]. We observed low levels of necrosis in two of the non-illuminated tumors and one of the control tumors indicating that spontaneous necrosis can potentially occur in these tumor types (Fig. 5a-b and Supplementary Fig. 2). However, as all five of our PDT-illuminated tumors showed some level of necrosis, whereas only one of our control tumors had a necrosis level less than 1%, the base level of necrosis at this tumor volume appeared to be minimal. With regard to the level of necrosis noted in two of the non-illuminated tumors, we could not definitively conclude whether this was necrotic formation due to the natural tendencies of this tumor type or if it was due to low levels of PDT-induced effects following general light exposure. The level of necrosis was greater in the non-illuminated tumors than in the controls, suggesting that this difference could be due to the latter. We remarked that the annotated necrotic areas from the H&E images showed patterns comparable to those of the parallel fluorescent slides and indicated a similar percentage of necrotic area; consequently, we concluded that 800CW Carboxylate strongly accumulated in necrotic regions (Fig. 5b-c and Supplementary Fig. 2). This specific accumulation of 800CW Carboxylate in necrotic regions was also observed in our previous studies using 800CW as a fluorescent and radiolabeled imaging agent [15, 16].

PDT treatment typically induces temporary edema and inflammation due to the disruption of the blood flow and by the production of ROS [3]. This can affect fluorescence due to the disruption of blood flow. Consequently, measuring fluorescence during this state can be affected by hemoglobin absorption. However, this effect should be minimal for the necrosis marker 800CW since most hemoglobin absorption occurs under 600 nm [28]. To overcome this potential barrier to hemoglobin absorption and for it to be translatable to the clinic, 800CW can be used as a contrast agent for SPECT/CT or PET/CT imaging. This can be accomplished by radiolabeling with indium-111 for SPECT-imaging and gallium-68 for PET imaging, as performed by our group previously in a breast cancer model [15, 29]. Using 800CW, radiolabeled with indium-111 or gallium-68 could be applied in the clinic for pre- and post-treatment assessment of pancreatic cancer. Computed tomography (CT) is one of the standard imaging modalities used for evaluating pancreatic cancer and has been used to assess PDT-induced post-treatment pancreatic necrosis [30]. For optimal assessment of changes in the tumor after treatment, it is important to take measurements both before and after PDT. This approach can also be used for monitoring response rates with the goal of assessing whether further treatment is needed. In the future, it would be interesting to investigate whether this necrosis marker can be used to distinguish responders from non-responders due to the presence of 800CW Carboxylate after PDT. Another application could be optoacoustic imaging if better spatial resolution is desired, because the absorption of 800CW Carboxylate is in the NIR spectrum range, which allows for deeper tissue penetration [31]. This necrosis imaging agent can easily be translated to other imaging modalities if increased resolution or clinical application is desired.

## Conclusions

In our study, we validated that fluorescent imaging can be applied for evaluating and assessing both pre- and post-PDT imaging agents in a preclinical context. Fluorescent imaging can be used to monitor the whole-body distribution of the PS Bremachlorin as well as for imaging necrotic cell death, using 800CW Carboxylate for rapidly assessing the effect of PDT; both of which have been performed in a medium-throughput and cost-effective manner.

### Supplementary Information

ESM 1 Supplementary Fig. 1 Representative fluorescence images of tumors, skins of tumors, spleens, pancreases, small intestines, stomachs, kidneys, livers and muscles *ex vivo* at 6 and 24 hours after Bremachlorin injection and the control group (that did not receive Bremachlorin). (PNG 256 kb)

ESM 2 Supplementary Fig. 2 All tumor tissues that exhibited necrosis (determined by a pathologist) in the control (*n*=1), PDT-non-illuminated (*n*=2), and PDT-illuminated (*n*=5) groups. 800CW Carboxylate fluorescence (left) and H&E-stained (right). H&E images showing the necrotic area (red annotation) and tumor outline (yellow annotation). All images in the figure are shown with scale bars of 1 mm. (PNG 1752 kb)

ESM 3 Supplementary Fig. 3 Days taken to reach desired tumor volume after injection of PDAC cells for (A) Bremachlorin uptake (*n*=4 per group) and for (B) Bremachlorin PDT group and control group (*n*=5). Average tumor volume on day of desired tumor volume for (C) Bremachlorin uptake (*n*=4) and for (D) Bremahclorin PDT group and control group (*n*=5). (PNG 83 kb)

## Data Availability

All relevant data is contained within the article. The original contributions presented in the study are included in the article or supplementary material. Further inquiries can be directed to the corresponding author.
