# Supplementary figures and images for: Rapid Assessment of Bio-distribution and Antitumor Activity of the Photosensitizer Bremachlorin in a Murine PDAC Model: Detection of PDT-induced Tumor Necrosis by IRDye® 800CW Carboxylate, Using Whole-Body Fluorescent Imaging
Source: Mol Imaging Biol. 2024 Jun 18;26(4):616–27. doi: 10.1007/s11307-024-01921-1 (PMC11281978; doi:10.1007/s11307-024-01921-1)

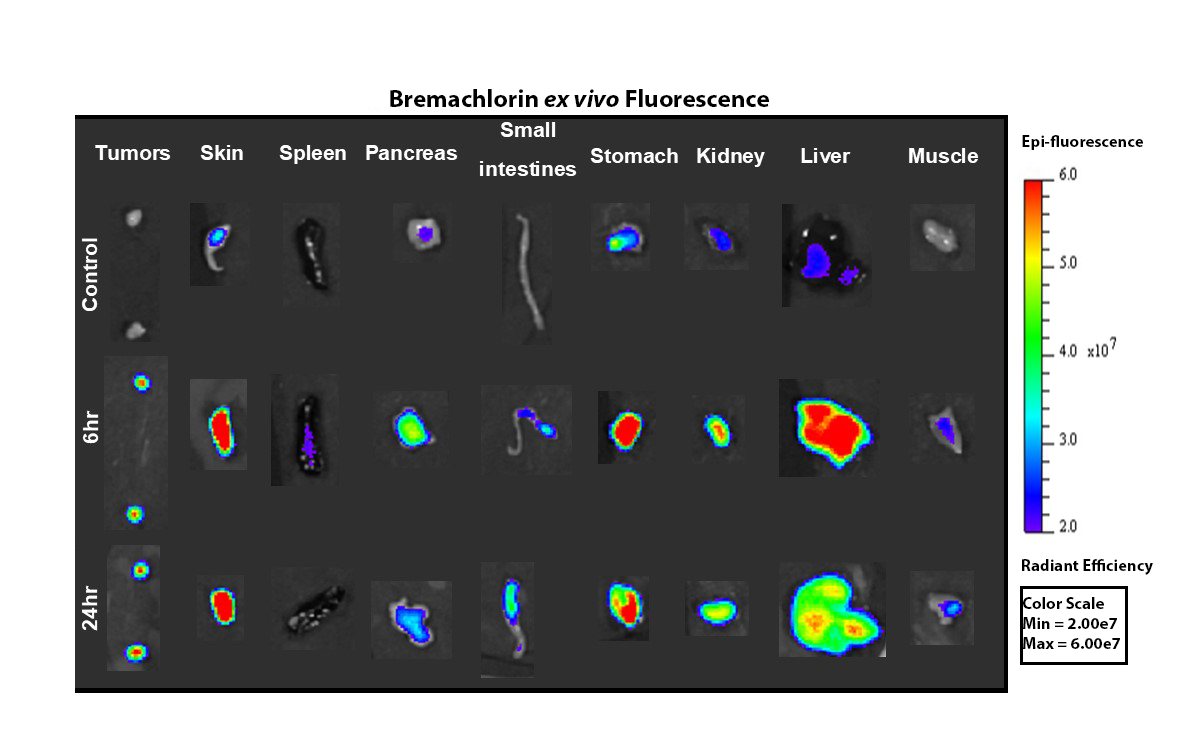

Supplement: Supplementary file 1 — Supplementary Fig. 1 Representative fluorescence images of tumors, skins of tumors, spleens, pancreases, small intestines, stomachs, kidneys, livers and muscles ex vivo at 6 and 24 hours after Bremachlorin injection and the control group (that did not receive Bremachlorin). (PNG 256 kb) [file 11307_2024_1921_MOESM1_ESM.png]

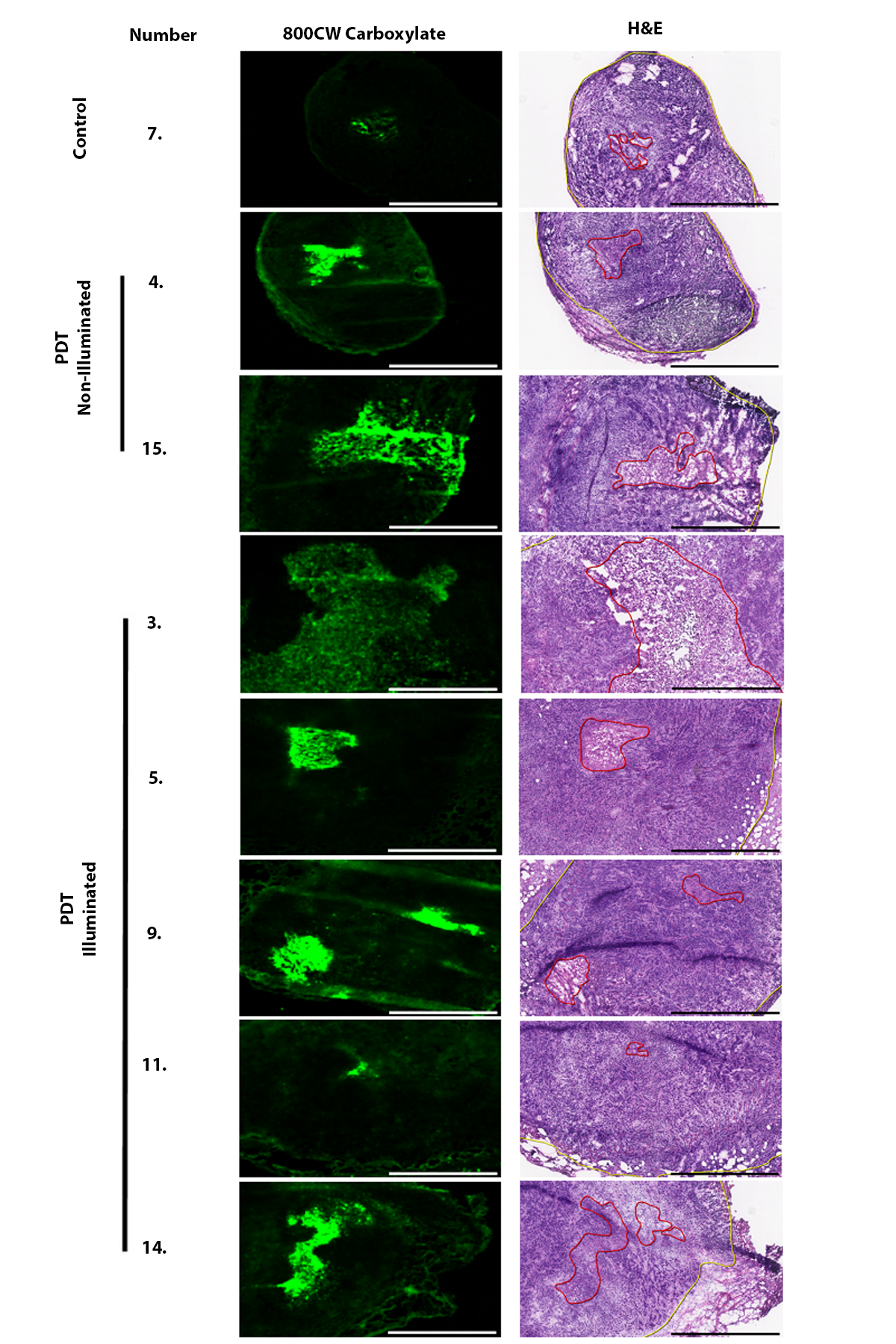

Supplement: Supplementary file 2 — Supplementary Fig. 2 All tumor tissues that exhibited necrosis (determined by a pathologist) in the control (n=1), PDT-non-illuminated (n=2), and PDT-illuminated (n=5) groups. 800CW Carboxylate fluorescence (left) and H&E-stained (right). H&E images showing the necrotic area (red annotation) and tumor outline (yellow annotation). All images in the figure are shown with scale bars of 1 mm. (PNG 1752 kb) [file 11307_2024_1921_MOESM2_ESM.png]

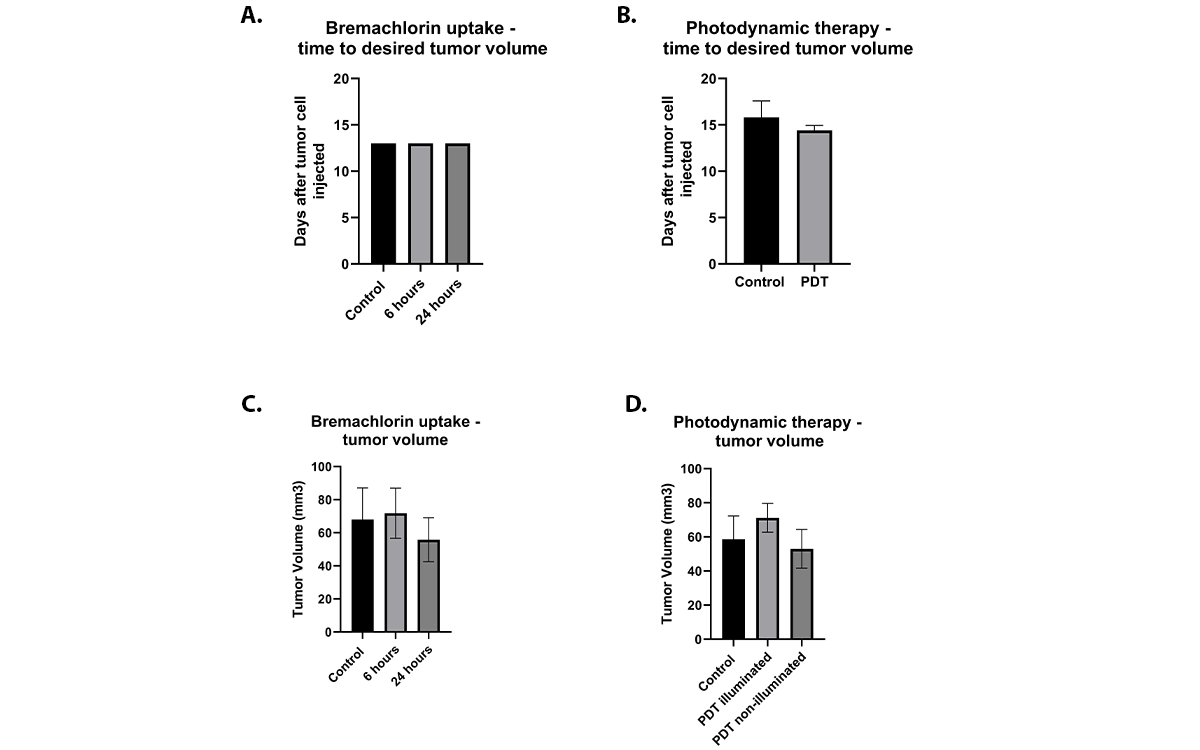

Supplement: Supplementary file 3 — Supplementary Fig. 3 Days taken to reach desired tumor volume after injection of PDAC cells for (A) Bremachlorin uptake (n=4 per group) and for (B) Bremachlorin PDT group and control group (n=5). Average tumor volume on day of desired tumor volume for (C) Bremachlorin uptake (n=4) and for (D) Bremahclorin PDT group and control group (n=5). (PNG 83 kb) [file 11307_2024_1921_MOESM3_ESM.png]
